# Development and Preliminary Mechanistic Evaluation of a Novel Liposomal QS-21 and CpG ODNs Adjuvant System for Enhancing Vaccine Immunogenicity

**DOI:** 10.3390/vaccines14060510

**Published:** 2026-06-05

**Authors:** Jun Ge, Kangwei Xu, Yong Cao, Jiaojiao Sun, Lili Guo, Lilong Sun, Ke Liu, Jinbiao Lu, Jianqiang Li, Yixuan Zhang

**Affiliations:** 1 School of Life Science and Biopharmaceutics, Shenyang Pharmaceutical University, Shenyang 110016, China; 2 Grand Theravac Life Science Nanjing Co., Ltd., Pangu Road, Nanjing 210032, Chinalijianqiang@theravac.cn (J.L.); 3 National Institutes for Food and Drug Control, Beijing 102629, China

**Keywords:** vaccine adjuvant, liposome, Th1-type immune response

## Abstract

Developing potent adjuvants is critical for enhancing vaccine efficacy, particularly for subunit antigens. **Background/Objectives**: This study evaluates a novel composite adjuvant system combining liposomal QS-21 and CpG ODNs to enhance vaccine-induced immunogenicity, particularly Th1-type cellular immunity. **Methods**: To mitigate QS-21’s hemolytic toxicity and ensure precision delivery, a stable liposomal formulation was developed. Mice models were established using varicella-zoster virus (VZV) glycoprotein E (gE) or ovalbumin (OVA) as antigens to evaluate humoral and cellular immune responses. **Results**: Immunization with gE protein formulated with this novel adjuvant synergistically triggered robust immune responses, outperforming single adjuvants and the combination of QS-21/MPL. Across broad dose ranges, it induced higher Th1-type cellular immunity and comparable humoral immunity relative to AS01_B_. Mechanistic studies revealed that the adjuvant significantly enhances the recruitment of dendritic cells (DCs), monocytes, and neutrophils to draining lymph nodes (dLNs) while upregulating co-stimulatory molecules CD40 and CD86 on DCs. Furthermore, the formulation triggered robust, transient increases in Th1-associated cytokines (IFN-γ, IL-12) and chemokines (CXCL9, CXCL10) across the injection site, serum, and dLNs. **Conclusions**: These findings indicate that the liposomal QS-21 and CpG ODNs system is a highly effective platform for promoting robust Th1-biased immunity, offering a promising adjuvant candidate and a solid experimental foundation for developing next-generation vaccines requiring potent cellular immunity.

## 1. Introduction

Vaccines, as a critical tool for preventing and controlling infectious diseases, function primarily by inducing specific and long-lasting immune protective responses in the body. However, most purified antigens (such as recombinant proteins, peptides, etc.) exhibit weak immunogenicity when administered alone, making it difficult to elicit sufficiently strong cellular and humoral immune responses [1,2], which fails to meet clinical prevention and control requirements. Vaccine adjuvants, as substances capable of non-specifically enhancing antigen immunogenicity and modulating the type of immune response, have become an indispensable component of modern vaccine development systems. Their evolution is closely linked to advancements in vaccine technology and plays a significant role in improving vaccine efficacy and expanding the applicable population (e.g., the elderly, immunocompromised individuals) [3,4,5].

The development of vaccine adjuvants dates to the early 20th century. The discovery of aluminum adjuvant in 1926 marks the beginning of modern vaccine adjuvant development. Due to its wide availability, ease of preparation, and favorable safety profile, it remains among the most used adjuvants in human vaccines. Aluminum adjuvants primarily work by adsorbing antigens to prolong their retention time at the injection site and by activating innate immune cells, thereby enhancing humoral immune responses. It is particularly effective in inducing IgG1 subclass antibody responses and is widely used in vaccines against diseases such as diphtheria and tetanus [6]. However, aluminum adjuvants mainly induce a T helper 2 (Th2)-type humoral immune response and have limited efficacy for vaccines requiring a T helper 1 (Th1)-type cellular immune response (e.g., clearing intracellular pathogens, anti-tumor immunity) [7]. With continuous advancements in immunological research, the development of vaccine adjuvants has gradually shifted from “non-specific stimulation” to “precise modulation of immune responses” to overcome the drawbacks of aluminum adjuvants.

Novel adjuvants have emerged, such as Toll-like receptor (TLR) agonists (e.g., CpG ODNs, MPL), saponin-based adjuvants (e.g., QS-21) and water-in-oil emulsion adjuvants (e.g., Freund’s adjuvant, MF59). CpG oligodeoxynucleotides (ODNs) are specific agonists for Toll-like receptor 9 (TLR9). They mimic the characteristic structure of pathogenic DNA, inducing both cellular and humoral immunity to enhance the host’s immune response [8,9,10,11]. The HBV vaccine HEPLISAV-B^®^, composed of CpG 1018 adjuvant and HBsAg antigen, developed by Dynavax, received FDA approval in 2017 [12]. Among these, QS-21, a saponin-based adjuvant extracted from the bark of the Chilean soapbark tree (Quillaja saponaria Molina) [13], possesses strong immune-activating capabilities. It promotes maturation of dendritic cells (DCs), enhances T-cell proliferation and cytokine secretion, and modulates both humoral and cellular immune responses [14,15]. It has demonstrated excellent adjuvant activity in several candidate vaccines [16]. The ligand of TLR4, lipopolysaccharide (LPS), primarily contains lipid A as its structurally active adjuvant component. Under weakly acidic conditions, lipid A can hydrolyze into monophosphoryl lipid A (MPL). The AS01 adjuvant system, containing QS-21 and MPL and developed by GlaxoSmithKline, has been applied in herpes zoster vaccines SHINGRIX^®^ and respiratory syncytial virus (RSV) vaccines AREXVY^®^, receiving FDA approval in 2019 and 2023, respectively. Although single TLR agonists (such as CpG ODNs and MPL) can induce a Th1-type response, their immunostimulatory effect is insufficient when used alone, and they are influenced by the body’s immune status, making it difficult to establish long-lasting immune memory.

To address the limitations of existing adjuvants, the development of novel adjuvants is advancing rapidly toward “multi-component synergy, precise modulation, high safety, and low cost.” The construction of “composite adjuvant systems” is now a key research focus and priority. By rationally combining adjuvant components with distinct mechanisms—such as saponins, TLR agonists, and lipid carriers—these composite adjuvants achieve more efficient synergistic effects.

This study focuses on the glycoprotein E (gE) of the varicella-zoster virus (VZV) and systematically investigates the effects of the single and combined use of adjuvants such as QS-21, CpG ODNs, and MPL. Using ovalbumin (OVA) as an antigen, the study also analyzed the mechanism by which the liposomal QS-21 and CpG ODNs adjuvant affects the recruitment of antigen-presenting cells (APCs) and cytokine secretion, confirming that the liposomal QS-21 and CpG ODNs adjuvant can serve as a novel candidate vaccine adjuvant.

## 2. Materials and Methods

### 2.1. Reagents

The VZV gE protein was manufactured by Grand Theravac Life Science Nanjing Co., Ltd. (Nanjing, China). CpG ODNs were obtained from Desert King International LLC (Chula Vista, CA, USA). QS-21 was purchased from Shanghai SynTheAll Pharmaceutical Co., Ltd. (Shanghai, China) and packaged into liposomes by Grand Theravac Life Science Nanjing Co., Ltd. MPL was purchased from Shanghai ZZBIO Co., Ltd. (Shanghai, China). Goat-anti-mouse IgG antibodies were purchased from Sigma-Aldrich (Saint Louis, MO, USA). Goat-anti-mouse IgG1/IgG2a antibodies were purchased from SouthernBiotech (Birmingham, AL, USA). The Mouse IFN-γ ELISpot Kit was purchased from BD Biosciences (Franklin Lakes, NJ, USA). The OVA protein was purchased from InVivoGen (San Diego, CA, USA). The Shingrix vaccine was purchased from GlaxoSmithKline (GSK) (London, UK). LEGENDplex™ Multiplex Assays were purchased from BioLegend (San Diego, CA, USA). Anti-Mouse CD3ε FITC, anti-Mouse CD19 FITC, anti-Mouse CD11b PerCP-Cy5.5, anti-Mouse Ly6C BV421, anti-Mouse CD40 BV480, and anti-Mouse CD86 PE-Cy7 were purchased from BD Biosciences. Anti-Mouse CD4 BV510, anti-Mouse CD8a FITC, anti-Mouse CD49b FITC, and anti-Mouse CD11c AF700 were purchased from BioLegend. Anti-Mouse CD3 AF700, anti-Mouse IFN-γ PE, anti-Mouse TNF-α eFluor450, anti-Mouse IL-2 APC, anti-Mouse Ly6G PE, anti-Mouse MHC II SB600, anti-Mouse F4/80 PE-eFluor610, anti-Mouse CD169 eFluor660, Live/Dead Near IR, and the 2.4G2 antibody were purchased from Thermo Fisher Scientific (Waltham, MA, USA).

### 2.2. Adjuvant Component Combination Study

A total of 48 female C57BL/6 mice aged 5 weeks (SPF grade) were randomly assigned into eight experimental groups. Mouse grouping and the immunization schedule are shown in Table 1.

At Week 6, the mice were euthanized, and serum and splenocytes were collected to assess immune response levels, including the number of IFN-γ-secreting SFC, the proportion of IFN-γ-, IL-2- and TNF-α-producing CD4^+^ T cells in splenocytes, and serum gE-specific IgG antibody levels.

### 2.3. Liposomal Vaccine Adjuvant Preparation of QS-21 and CpG Adjuvant

The liposome adjuvant was prepared by the classic thin-film dispersion method. In brief, blank liposome is composed of phospholipid and cholesterol which were prepared using a series of processes such as membrane formation, hydration, homogenization, and extrusion. Subsequently, appropriate QS-21 and CpG ODNs solutions were added in sequence and mixed with the blank liposome at room temperature to obtain the liposomal QS-21/CpG adjuvant. The composite adjuvant was stored at 4 °C.

### 2.4. Study on Dose Ratio Optimization Between Liposomal QS-21 and CpG ODNs

A total of 42 female C57BL/6 mice aged 5 weeks (SPF grade) were used to investigate the dose ratio of liposomal QS-21 and CpG ODNs adjuvants, divided into 7 groups with 6 mice per group. Mouse grouping and the immunization schedule are shown in Table 2.

At Week 3, the mice were euthanized, and serum and splenocytes were collected to assess immune response levels, including the number of IFN-γ-secreting SFC (at a density of 1 × 10^5^ cells per well, and the data are finally presented converted to 1 × 10^6^ cells), the proportion of IFN-γ-, IL-2- and TNF-α-producing CD4^+^ T cells in splenocytes, and serum gE-specific IgG/IgG1/IgG2a antibody levels.

### 2.5. Effect of the Liposomal QS-21 and CpG ODNs Adjuvant on APCs Recruitment in Murine dLNs

Twenty-four female C57BL/6 mice aged 5 weeks (SPF grade) were randomly divided into four groups, with 6 mice per group. Mouse grouping and the immunization schedule are shown in Table 3.

At 24 h post-immunization, iliac lymph nodes were harvested and subjected to flow cytometry analysis to quantify DCs, monocytes, neutrophils, macrophages, and CD169^+^ macrophages in the lymph nodes.

### 2.6. Effect of the Liposomal QS-21 and CpG ODNs Adjuvant Combined with OVA Antigen on APCs Recruitment in Murine dLNs

Nine female C57BL/6 mice aged 5 weeks (SPF grade) were randomly divided into three groups, with 3 mice per group. Mouse grouping and the immunization schedule are shown in Table 4.

At 24 h post-immunization, iliac lymph nodes were harvested and subjected to flow cytometry analysis to quantify DCs, monocytes, neutrophils, and macrophages in the lymph nodes.

### 2.7. Effect of the Liposomal QS-21 and CpG ODNs Adjuvant Combined with OVA Antigen on Cytokine Levels in Murine dLNs, Injection Sites and Sera

Seventy-two female C57BL/6 mice aged 8 weeks (SPF grade) were randomly divided into three groups, with 24 mice per group. Mouse grouping and the immunization schedule are shown in Table 5.

At 3 h, 6 h, 24 h, and 48 h post-immunization, blood samples, iliac lymph nodes, and injection site muscle tissues were collected for analysis of cytokine expression levels by cytometric bead array (CBA).

### 2.8. ELISA

Mouse blood was collected in 1.5 mL microcentrifuge tubes, allowed to stand at 37 °C for 40 min, and then centrifuged at 4 °C and 12,000 rpm for 10 min to separate the serum. The supernatant was carefully transferred to a new 1.5 mL microcentrifuge tube and stored at −20 °C for subsequent use. A 96-well microplate was coated with gE protein and incubated overnight at 4 °C. After blocking with 2% skim milk in PBST, serially diluted serum samples were added. Following incubation with primary and secondary antibodies, TMB substrate was added, and the optical density (OD) value was measured at 450 nm with a reference wavelength of 630 nm.

### 2.9. ELISpot

Under aseptic conditions, the mouse spleen was harvested and ground through a strainer to prepare a single-cell suspension. Red blood cells were lysed using red blood cell lysis buffer, followed by centrifugation to collect splenocytes, which were then counted and adjusted to the desired concentration. An ELISpot plate pre-coated with anti-mouse IFN-γ capture antibody was blocked, and then each well was seeded with the adjusted splenocytes along with corresponding stimuli. The plate was incubated for 48–50 h at 37 °C in a 5% CO_2_ incubator. After incubation, the cell suspension was discarded, and the plate was washed with wash buffer. Biotinylated anti-mouse IFN-γ detection antibody was added and incubated at room temperature for 2 h. Following another wash, HRP-conjugated streptavidin was added and incubated at room temperature for 1 h. After a final wash, AEC substrate solution was added for color development. The reaction was terminated with stop solution once distinct spots emerged, and the spots in each well were counted using an ELISpot analyzer.

### 2.10. Flow Cytometry

Cells were resuspended in PBS containing 1% BSA and first incubated with 2.4G2 antibody for 5 min to block Fc receptors, and then each experiment involved staining with a distinct antibody panel.

(1) anti-Mouse CD3 AF700, anti-Mouse CD4 BV510, anti-Mouse CD8a FITC, anti-Mouse IFN-γ PE, anti-Mouse TNF-α eFluor450, anti-Mouse IL-2 APC and Live/Dead Near IR;
(2) anti-Mouse lineage-FITC Cocktail (CD3ε, CD19, CD49b), anti-Mouse Ly6G PE, anti-Mouse CD11c AF700, anti-Mouse MHC II SB600, anti-Mouse CD11b PerCP-Cy5.5, anti-Mouse F4/80 PE-eFluor610, anti-Mouse CD169 eFluor660, anti-Mouse Ly6C BV421, and Live/Dead Near IR;
(3) anti-Mouse lineage-FITC Cocktail (CD3ε, CD19, CD49b), anti-Mouse Ly6G PE, anti-Mouse CD11c AF700, anti-Mouse MHC II SB600, anti-Mouse CD11b PerCP-Cy5.5, anti-Mouse F4/80 PE-eFluor610, anti-Mouse Ly6C BV421, anti-Mouse CD40 BV480, anti-Mouse CD86 PE-Cy7 and Live/Dead Near IR.

Fluorescent signals were acquired using an Attune NxT flow cytometer (Thermo Fisher Scientific) and analyzed with FlowJo software v10 (BD Biosciences). DCs were gated as LIN^−^CD11c^+^MHC II^+^, monocytes as LIN^−^CD11b^+^Ly6C^+^Ly6G^−^F4/80^−^, macrophages as LIN^−^F4/80^+^, and neutrophils as LIN^−^CD11b^+^Ly6CintLy6G^+^F4/80int (Figure S2).

### 2.11. Cytometric Bead Array (CBA)

The experiment was performed using the LEGENDplex™ MU Cytokine Release Syndrome Panel (Cat.741023, BioLegend) strictly according to the manufacturer’s protocol. Provided standards were serially diluted and co-incubated with premixed beads on a shaker for 2 h. After centrifugation, biotinylated detection antibodies were added and incubated with shaking for 1 h. Streptavidin-PE conjugate antibody was added and incubated with shaking for 30 min. Following centrifugation of the mixture, the pellet was washed once with assay buffer. After recentrifugation, cells were resuspended and acquired on a flow cytometer. Cloud-based LEGENDplex™ Data Analysis Software (BioLegend) was used to generate standard curves and quantify samples. For sample testing, standards were replaced with test supernatants and processed identically. Cytokine concentrations were calculated by interpolating sample MFI values against the standard curve.

### 2.12. Statistical Analysis

Antibody titers were log-transformed (log10) to achieve a normal distribution for statistical analysis. Data are presented as log-transformed geometric mean titers (GMTs). Statistical analysis was performed with GraphPad Prism software v10. Statistical significance was determined with nonparametric one-way ANOVA. *: *p* < 0.05; **: *p* < 0.01; ***: *p* < 0.001; ****: *p* < 0.0001.

## 3. Results

### 3.1. Potent Synergistic Immunostimulatory Effects of Combined QS-21 and CpG ODNs Adjuvant

**Figure 1:**
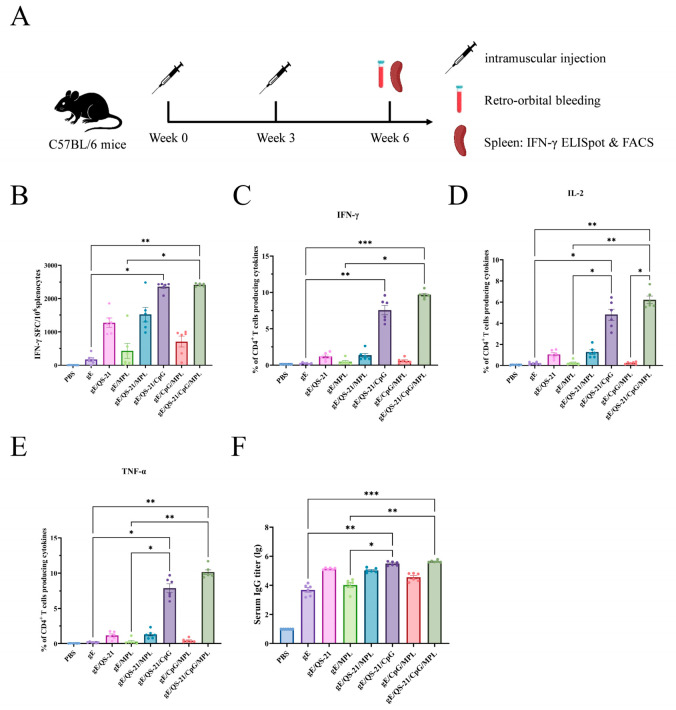
Immune response levels in mice immunized with gE protein combined with different adjuvants (*n* = 6, Mean ± SEM). (**A**) Schematic of the immunization schedule; (**B**) gE-specific IFN-γ-secreting splenocytes measured by ELISpot. (**C**–**E**) Frequency of multifunctional CD4^+^ T cell subsets secreting IFN-γ, TNF-α, and IL-2 analyzed by flow cytometry. (**F**) Serum levels of gE-specific IgG, values are expressed as the mean ± SEM of log10-transformed titers. Kruskal–Wallis test with Dunn’s post hoc test; *: *p* < 0.05, **: *p* < 0.01, ***: *p* < 0.001.

Three weeks after the second immunization, mice in the PBS group did not induce a gE-specific cellular immune response. Compared with mice immunized with gE protein alone, the groups of mice immunized with gE protein combined with different adjuvants all showed increased levels of gE-specific cellular immune responses. However, only the gE/QS-21/CpG ODNs group and the gE/QS-21/CpG ODNs/MPL group showed statistically significant differences (*p* < 0.05, *p* < 0.01, respectively). The gE/QS-21/CpG ODNs group and the gE/QS-21/CpG ODNs/MPL group both reached the upper detection limit (i.e., 2500 SFC/10^6^ cells) and their responses were higher than those of the groups immunized with gE protein formulated with single adjuvants (QS-21, MPL) or dual adjuvants (QS-21/MPL, CpG ODNs/MPL) (Figure 1B). Among them, the groups without QS-21, namely the gE/MPL group and gE/CpG/MPL group, showed a relatively small increase, indicating that QS-21 is crucial for enhancing the levels of these cytokines. The gE/QS-21/MPL group showed a limited increase compared with the gE/QS-21 group, suggesting that the addition of MPL had a limited effect on top of QS-21. In contrast, the gE/QS-21/CpG group showed a substantial increase compared with the gE/QS-21 group, indicating a strong synergistic effect of CpG with QS-21. Furthermore, the gE/QS-21/CpG ODNs/MPL group showed increases in the levels of IFN-γ, IL-2, and TNF-α compared with the gE/QS-21/CpG ODNs group, but the extent of the increase was limited (Figure 1C–E). Mice in the PBS control group did not induce gE-specific IgG antibodies in serum. Compared with mice immunized with gE protein alone, all groups immunized with gE protein formulated with different adjuvants showed an increase in serum levels of gE-specific IgG antibodies. However, only the gE/QS-21/CpG ODNs group and the gE/QS-21/CpG ODNs/MPL group showed statistically significant differences (*p* < 0.01, *p* < 0.001, respectively). The gE-specific IgG antibody levels for the gE/QS-21/CpG ODNs/MPL group and the gE/QS-21/CpG ODNs group were 105.49 and 105.64, respectively, which are very close, indicating that the addition of MPL had a minimal impact (Figure 1F). Overall, the combination of CpG and QS-21 can exert a potent immunostimulatory effect, which is significantly stronger than that of QS-21 alone and superior to the combination of MPL and QS-21.

### 3.2. Preparation and Characterization of the Liposomal QS-21 and CpG ODNs Adjuvant

**Figure 2:**
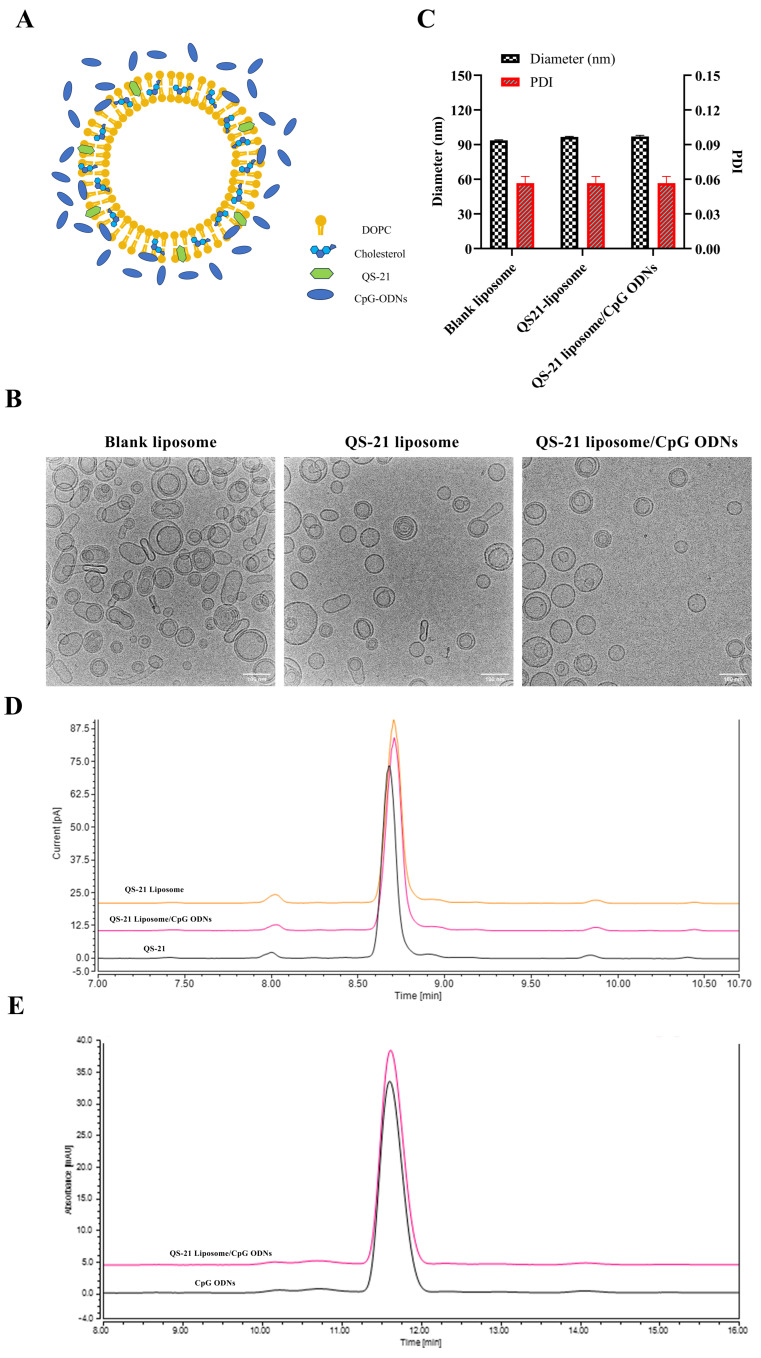
Characterization of the liposomal QS-21/CpG ODNs adjuvant system. (**A**) Schematic diagram of liposomal QS-21/CpG ODNs adjuvant system. (**B**) TEM observations of blank liposome, QS-21 liposome, and liposomal QS-21/CpG ODNs adjuvant in the process by cryo-electron micrographs, scale bar: 100 nm. (**C**) Diameter and PDI of blank liposome, QS21–liposome, and liposomal QS-21/CpG ODNs adjuvant in the process by dynamic light scattering (DLS). (**D**) Comparison of QS21 relevant substances for QS-21 (black), QS-21 liposome (orange), and liposomal QS-21/CpG ODNs adjuvant (pink) in the process by HPLC. (**E**) Comparison of CpG ODNs relevant substances for CpG ODNs (black) and liposomal QS-21/CpG ODNs adjuvant (pink) in the process by HPLC.

To eliminate the hemolytic toxicity (Figure S1) of QS-21 and achieve precision delivery, we developed a composite adjuvant system by encapsulating QS-21 into liposomes and combining them with CpG ODNs (the schematic is shown in Figure 2A). Subsequently, cryo-electron microscopy (cryo-EM) and dynamic light scattering (DLS) were employed to characterize the particle size distribution and morphology of the liposomal QS-21/CpG ODN adjuvant. Spherical or quasi-spherical particles with bilayer lipid membrane structures were observed in blank liposomes, QS-21 liposomes, and the composite liposomal adjuvant (Figure 2B). The average particle sizes for blank liposomes, QS-21 liposomes, and the composite adjuvant were 94 ± 1 nm, 97 ± 1 nm, and 97 ± 1 nm, respectively, with corresponding polydispersity indices (PDI) of 0.06 ± 0.01, 0.06 ± 0.01, and 0.06 ± 0.01 (Figure 2C). Furthermore, high-performance liquid chromatography (HPLC) was used to characterize the formulated products to ensure no alterations occurred in QS-21 or CpG ODNs. No significant related substances or degradation products were observed after the addition of QS-21 or CpG ODNs (Figure 2D,E). These results demonstrate that the liposomal QS-21/CpG ODN composite adjuvant system exhibits exceptional structural and chemical stability, and the preparation process has no adverse impact on either the carrier structure or the adjuvant components.

### 3.3. Synergistic Enhancement of Th1 Immunity by Liposomal QS-21 and CpG ODNs Across a Broad Dose Range

**Figure 3:**
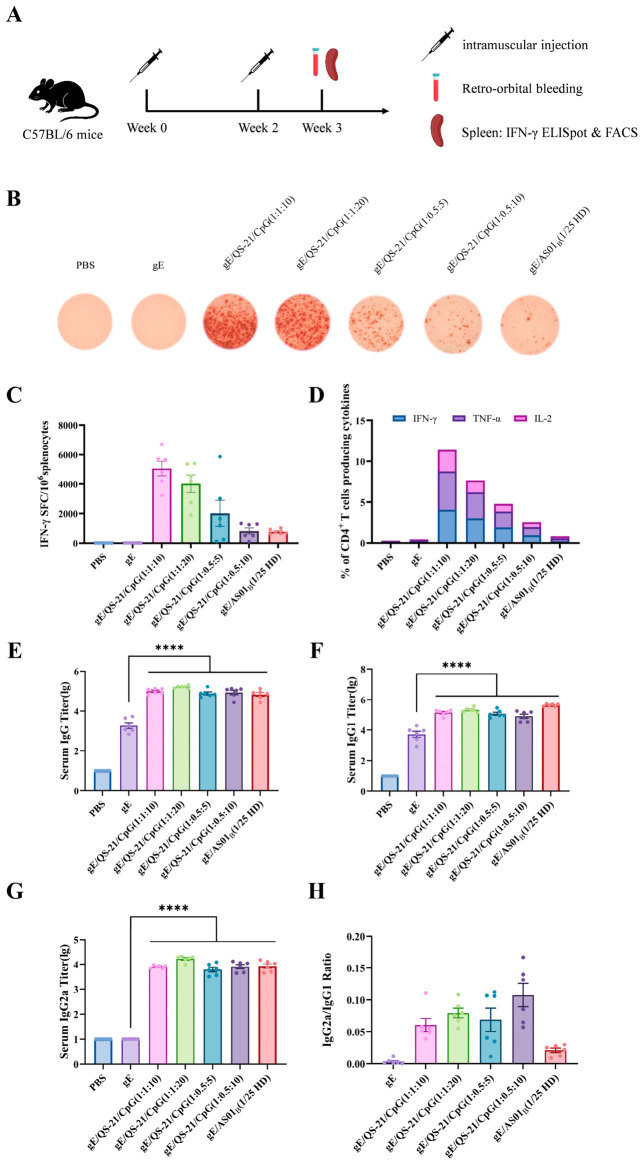
Immune response levels in mice immunized with various dosage combinations of gE antigen, liposomal QS-21, and CpG ODNs. Mice (*n* = 6 per group) were immunized with the different vaccine formulations. (**A**) Schematic of the immunization schedule. (**B**) Representative images of IFN-γ-secreting splenocytes (at a density of 1 × 10^5^ cells per well, and the data are finally presented converted to 1 × 10^6^ cells). (**C**) gE-specific IFN-γ-secreting splenocytes measured by ELISpot. (**D**) Frequency of multifunctional CD4^+^ T cell subsets secreting IFN-γ, TNF-α, and IL-2 analyzed by flow cytometry. (**E**–**G**) Serum levels of gE-specific IgG, IgG1, and IgG2a. (**H**) The ratio of IgG2a to IgG1 antibodies in serum. Data are presented as Mean ± SEM. One-way ANOVA with Tukey’s post hoc test; ****: *p* < 0.0001.

To explore the dose ranges of liposomal QS-21 and CpG ODNs, a repeated immunization mice model with gE protein in mice was established using the gE protein formulated with various ratios of the composite adjuvant. A negative group, a gE protein-alone immunization group, and a positive group immunized with gE protein formulated with a 1/25 human dose of AS01_B_ were included. The results showed that, compared with the gE protein-alone immunization group, all four different doses of the QVA/liposomal QS-21/CpG ODNs combination significantly increased the level of gE-specific IFN-γ-secreting splenocytes, and all were higher than those in group G, which received 0.04 human dose of Shingrix (Figure 3C). Further analysis of CD4^+^ T cell subsets secreting IFN-γ, TNF-α, and IL-2 by flow cytometry yielded consistent results with the ELISpot assay: compared with the gE protein-alone immunization group, all four different doses of the QVA/liposomal QS-21/CpG ODNs combination significantly increased the proportion of CD4^+^ T cells secreting IFN-γ, TNF-α, and IL-2, and all were higher than those in group G (Figure 3D). In terms of humoral immunity, there were no significant differences in serum IgG, IgG1, and IgG2a antibody levels among the groups immunized with gE combined with four different adjuvant dose combinations, nor were there significant differences compared with group G. However, all these groups showed a significant increase compared with the gE-alone immunization group (Figure 3E–G). Compared with group B, the IgG2a/IgG1 ratio was significantly higher in groups C-G. Additionally, the groups immunized with gE combined with liposomal QS-21 and CpG ODNs also showed a higher ratio than group G (Figure 3H). In summary, the combination of CpG ODNs with liposomal QS-21 at multiple doses, compared with MPL combined with liposomal QS-21, induced a higher level of cellular immunity and a comparable level of humoral immunity, while better promoting a Th1-type immune response.

### 3.4. Recruitment and Activation of Innate Immune Cells in dLNs by Liposomal QS-21 and CpG ODNs Adjuvant

**Figure 4:**
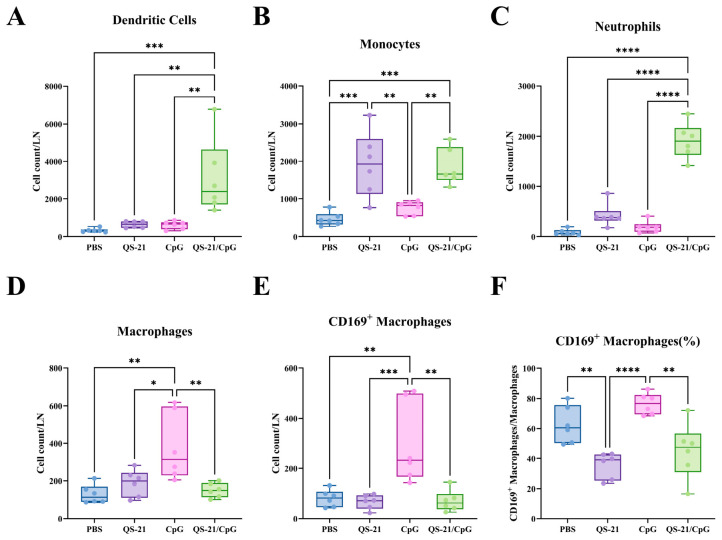
Immune cell levels in iliac lymph nodes of mice 24 h post-immunization (*n* = 6 per group, horizontal line represents median). (**A**) Absolute numbers of DCs. (**B**) Absolute numbers of monocytes. (**C**) Absolute numbers of neutrophils. (**D**) Absolute numbers of macrophages. (**E**) Absolute numbers of CD169^+^ macrophages. (**F**) Proportion of CD169^+^ macrophages among total macrophages. One-way ANOVA with Tukey’s post hoc test; *: *p* < 0.05, **: *p* < 0.01, ***: *p* < 0.001, ****: *p* < 0.0001.

Compared with the PBS group, both liposomal QS-21 and CpG ODNs groups showed increased absolute numbers of DCs in mouse iliac lymph nodes, though without statistical significance. The liposomal QS-21/CpG ODNs adjuvant group exhibited a significant increase in DCs numbers compared with all other three groups (Figure 4A). CpG ODNs group demonstrated elevated monocytes counts versus PBS group without significant difference, whereas both liposomal QS-21 and liposomal QS-21/CpG ODNs adjuvant groups showed significantly increased monocytes numbers compared with PBS and CpG ODNs groups (Figure 4B). Neutrophils count increased in liposomal QS-21 and CpG ODNs groups relative to PBS group without significance. Liposomal QS-21/CpG ODNs adjuvant group displayed significantly higher neutrophil numbers than all other groups (Figure 4C). Liposomal QS-21 and liposomal QS-21/CpG ODNs adjuvant groups had non-significantly increased macrophage counts compared with PBS group. CpG ODNs group showed significantly elevated macrophages numbers versus all other groups. Liposomal QS-21 and liposomal QS-21/CpG ODNs adjuvant groups exhibited non-significant decreases in CD169^+^ macrophages count relative to PBS group (Figure 4D). CpG ODNs group demonstrated significantly increased absolute numbers of CD169^+^ macrophages compared with other groups. The proportion of CD169^+^ macrophages among total macrophages increased in CpG ODNs group versus PBS group without significance (Figure 4E). Both liposomal QS-21 and liposomal QS-21/CpG ODNs adjuvant groups showed decreased proportions of CD169^+^ macrophages relative to PBS group, with QS-21 group reaching statistical significance. Compared with CpG ODNs group, liposomal QS-21, and liposomal QS-21/CpG ODNs adjuvant groups exhibited significantly reduced proportions of CD169^+^ macrophages (Figure 4F).

### 3.5. Liposomal QS-21 and CpG ODNs Adjuvant Enhances APC Recruitment in dLNs and Upregulates CD40 and CD86 on DCs

**Figure 5:**
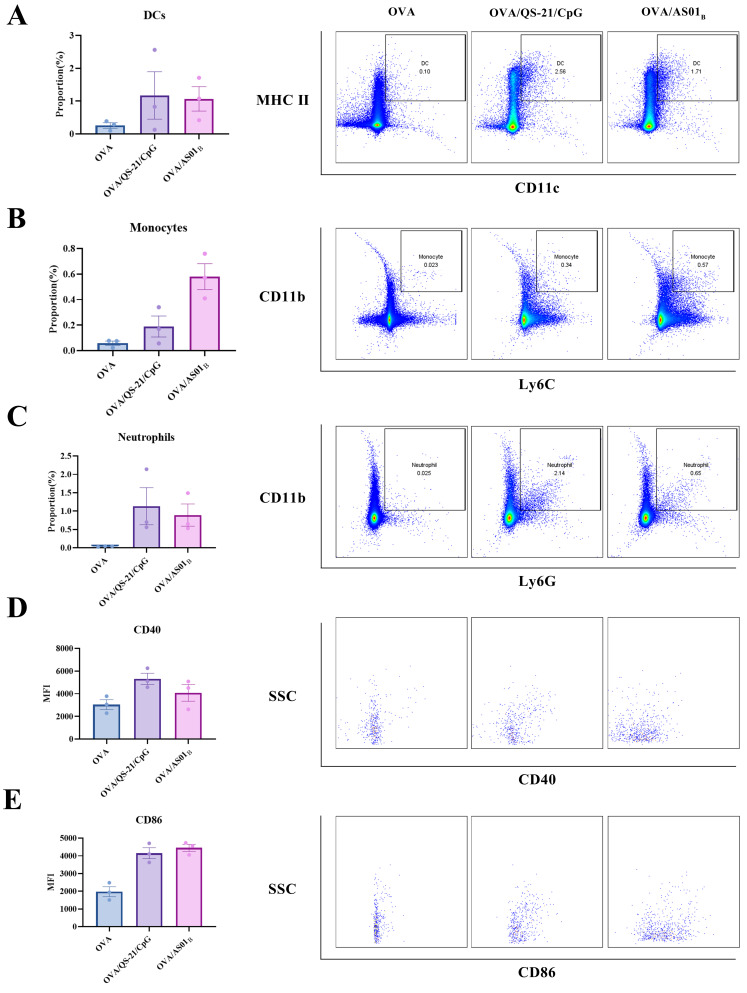
Levels of DCs, monocytes, and neutrophils in mouse inguinal lymph nodes 24 h post-immunization with OVA antigen (*n* = 3 per group, Mean ± SEM). (**A**) Proportion of DCs in Lin^−^ cells. (**B**) Proportion of monocytes in Lin^−^ cells. (**C**) Proportion of neutrophils in Lin^−^ cells. (**D**) Mean fluorescence intensity (MFI) of CD40 on DCs. (**E**) MFI of CD86 on DCs. Kruskal–Wallis test with Dunn’s post hoc test.

Twenty-four hours after immunization, the proportion of DCs among all Lin^−^ (CD3^−^CD19b^−^CD49b^−^) cells in the iliac lymph nodes of mice in the OVA protein-only group without adjuvant was 0.260%. In comparison, the proportions of DCs among all Lin^−^ cells in the lymph nodes of mice immunized with OVA formulated with liposomal QS-21/CpG ODNs adjuvant or AS01_B_ both increased, reaching 1.173% and 1.070%, respectively, with comparable levels between the two groups (Figure 5A). The proportion of monocytes among all Lin^−^ cells in the iliac lymph nodes of the OVA protein-only group without adjuvant was 0.058%. In contrast, the proportions of monocytes among all Lin^−^ cells in the lymph nodes of mice immunized with OVA formulated with liposomal QS-21/CpG ODNs adjuvant or AS01_B_ increased to 0.189% and 0.580%, respectively, with the OVA/liposomal QS-21/CpG ODNs adjuvant group showing a lower level than the OVA/AS01_B_ group (Figure 5B). The proportion of neutrophils among all Lin^−^ cells in the iliac lymph nodes of the OVA protein-only group without adjuvant was 0.036%. By comparison, the proportions of neutrophils among all Lin^−^ cells in the lymph nodes of mice immunized with OVA formulated with liposomal QS-21/CpG ODNs adjuvant or AS01_B_ increased to 1.133% and 0.890%, respectively, with the OVA/liposomal QS-21/CpG ODNs adjuvant group exhibiting a higher level than the OVA/AS01_B_ group (Figure 5C). In contrast, the proportion of macrophages among all Lin^−^ cells in the lymph nodes of mice immunized with OVA formulated with liposomal QS-21/CpG ODNs adjuvant or AS01_B_ remained largely unchanged compared to the OVA protein-only group without adjuvant, showing no significant differences. These results were consistent with those observed in the absence of OVA antibody but with adjuvant stimulation alone (Figure 4), indicating that liposomal QS-21/CpG ODNs adjuvant indeed enhances the recruitment of antigen-presenting cells (APCs) in the mouse lymph nodes. Furthermore, compared to the OVA protein-only group without adjuvant, the mean fluorescence intensity (MFI) of CD40 and CD86 co-stimulatory molecules on the surface of DCs in the lymph nodes of mice immunized with OVA formulated with liposomal QS-21/CpG ODNs adjuvant or AS01_B_ significantly increased (Figure 5D), demonstrating that both the liposomal QS-21/CpG ODNs adjuvant and AS01_B_ substantially promote the expression of CD40 and CD86 co-stimulatory molecules on the surface of DCs.

### 3.6. Liposomal QS-21 and CpG ODNs Adjuvant Enhances Cytokine and Chemokine Responses in dLNs, Injection Site, and Serum

Compared with mice immunized with OVA alone without adjuvant, the liposomal QS-21/CpG ODNs adjuvant group showed a significant increase in IFN-γ levels post-immunization. IFN-γ peaked at 6 h (339 pg/mL) and declined to the 3 h level by 24 h in dLNs. In serum and injection site, levels peaked at 24 h (311 pg/mL and 25 pg/mL, respectively) and nearly returned to the 3 h level by 48 h. The OVA/AS01_B_ group exhibited similar kinetic trends across all three tissues: IFN-γ peaked in lymph nodes at 6 h (505 pg/mL, higher than QS-21/CpG adjuvant), in serum at 6 h (326 pg/mL, unlike the 24 h peak with QS-21/CpG adjuvant), and at injection site at 24 h (60 pg/mL, higher than QS-21/CpG adjuvant).

IL-12 levels were significantly elevated in the OVA/liposomal QS-21/CpG ODNs adjuvant group compared with both the OVA-alone and OVA/AS01_B_ groups. Peaks occurred at 6 h in dLNs (20 pg/mL), serum (59 pg/mL), and injection site (4 pg/mL), declining to near 3 h levels by 24 h. Levels in dLNs and serum were much higher than at injection site. At 3 h, IL-12 at the injection site was like the other groups, but significantly higher in dLNs and serum.

CXCL9 expression increased markedly in both OVA/liposomal QS-21/CpG ODNs adjuvant and OVA/AS01_B_ groups compared with OVA-alone group. In dLNs, it peaked at 6 h (20 pg/mL, 10,472 pg/mL, and 8856 pg/mL, respectively) and returned to near baseline by 48 h. Serum levels remained elevated through 48 h without fully returning to baseline (593 pg/mL, 254 pg/mL). At injection site, levels were near baseline at 6 h, peaked at 24 h (2102 pg/mL, 1744 pg/mL), and returned to baseline by 48 h.

CXCL10 levels also rose significantly in the OVA/liposomal QS-21/CpG ODNs adjuvant and OVA/AS01_B_ groups. In dLNs, levels were near baseline at 6 h and peaked at 24 h (32,205 pg/mL, 25,946 pg/mL), returning to baseline by 48 h. In serum, the OVA/liposomal QS-21/CpG ODNs adjuvant group peaked at 24 h (11,818 pg/mL) and remained above baseline at 48 h, while the OVA/AS01_B_ group peaked earlier at 6 h (4647 pg/mL, markedly lower than OVA/QS-21/CpG adjuvant group) and returned to baseline by 48 h. At injection sites, both groups peaked at 24 h (2872 pg/mL, 1866 pg/mL) and returned to baseline by 48 h.

Compared with OVA-alone and OVA/AS01_B_ groups, the OVA/liposomal QS-21/CpG ODNs adjuvant group had significantly higher IL-4 levels in dLNs, peaking at 6 h (6 pg/mL) and declining to near baseline by 24 h. In serum, IL-4 levels at 24 h were significantly lower in both adjuvanted groups than in the OVA-alone group. At injection site, the OVA/liposomal QS-21/CpG ODNs adjuvant and OVA-alone groups showed similar kinetics: levels peaked at 6 h (0.89 pg/mL, 0.56 pg/mL) and steadily decreased to baseline by 48 h. The OVA/AS01_B_ group peaked at 3 h (0.88 pg/mL) and declined to baseline by 24 h.

**Figure 6:**
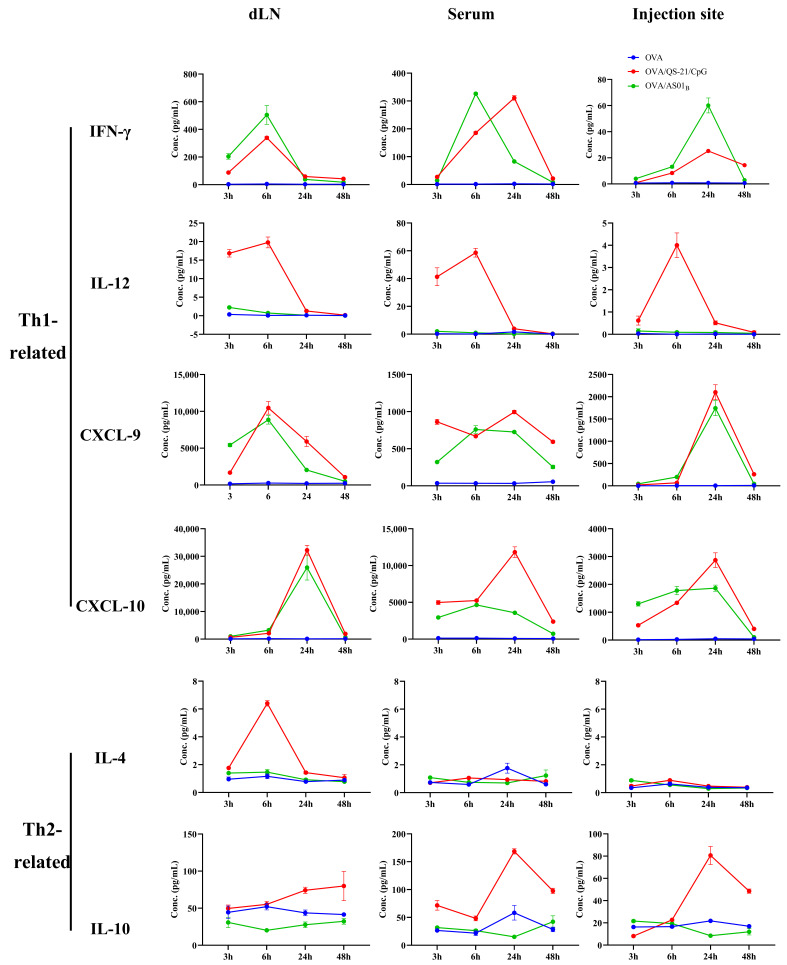
Cytokine levels at indicated time points post-immunization with OVA antigen in mice (*n* = 3/group, Mean ± SEM).

IL-10 expression in the OVA/liposomal QS-21/CpG ODNs adjuvant group was significantly higher compared with the other two groups. In dLNs, it increased steadily from 3 h to 48 h. In serum and injection site, IL-10 peaked at 24 h (169 pg/mL, 80 pg/mL) and remained high at 48 h without returning to baseline (Figure 6).

## 4. Discussion

This study is the first to demonstrate that a novel composite adjuvant composed of liposomal QS-21 and CpG ODNs can synergistically induce robust immune responses in mice, reaching a level comparable to that of the clinically established adjuvant AS01_B_. The liposomal QS-21/CpG ODNs adjuvant significantly enhances the recruitment of APCs to the dLNs and their expression of co-stimulatory molecules, while shaping a cytokine environment rich in IL-12, CXCL9, and CXCL10, thereby efficiently promoting antigen-specific T-cell immunity and high-titer antibody production.

The core finding of this study is the exceptional synergistic effect exhibited by the combination of QS-21 and CpG ODNs. In a study investigating adjuvant compositions, the gE protein alone induced only a weak immune response. However, the group immunized with gE combined with QS-21 and CpG adjuvant showed significantly higher levels of gE-specific IFN-γ ELISpot responses and IFN-γ, IL-2, and TNF-α secretion by CD4^+^ T cells compared with the gE-alone group (*p* < 0.01) and groups immunized with gE combined with either QS-21 or CpG alone. These responses even reached the detection limit, highlighting the strong synergy between QS-21 and CpG ODNs. Notably, the addition of MPL to this combination did not provide significant additional benefit, so there is no need—and it is inadvisable—to introduce MPL. Thus, the novel composite adjuvant has been defined as consisting of QS-21 and CpG ODNs.

However, QS-21 still exhibits toxicity in humans [12,13,17]. Studies on GSK’s AS-series adjuvants have demonstrated that liposomal formulations of QS-21 can significantly reduce the incidence of notable events in rat skeletal muscle [18]. Therefore, we further optimized QS-21 and CpG adjuvant by formulating it into a liposomal form. The results showed that liposomal QS-21 and CpG ODNs adjuvant possess favorable physicochemical properties (uniform particle size, regular morphology) and component compatibility. It is noteworthy that the introduction of QS-21 led to a slight increase in liposome particle size, while the subsequent addition of CpG ODNs did not cause a further size change. This phenomenon aligns with the known characteristics of saponin adjuvant interactions with liposomes: QS-21, as an amphiphilic saponin, has a hydrophobic portion that can embed into the lipid bilayer, while its hydrophilic sugar chains are exposed on the surface. This insertion may cause slight membrane expansion or structural reorganization, leading to the observed size increase [19]. The absence of further size change upon adding CpG ODNs suggests good physical compatibility with the QS21–liposome complex. CpG ODNs is likely associated with the liposomes via surface adsorption or internal encapsulation without disrupting the overall structure [20], thus providing a structural basis for their synergistic effects in immune activation.

Notably, liposomal QS-21 combined with CpG ODNs stimulated humoral immune responses comparable to those induced by AS01_B_ across multiple dosage combinations, and elicited higher levels of Th1-type cellular immune responses than AS01_B_. This is particularly crucial for clearing intracellular pathogens, such as herpesviruses and Mycobacterium tuberculosis.

To further investigate the mechanism of action of the liposomal QS-21 and CpG ODNs adjuvant, we conducted a series of experiments using the antigen OVA. The results showed that compared with AS01_B_ (which contains liposomal QS-21 and MPL), liposomal QS-21 and CpG ODNs adjuvant induced significantly higher levels of IL-12, a key cytokine driving Th1-type immune response differentiation [21,22]. Furthermore, the liposomal QS-21/CpG ODNs adjuvant induced higher and more sustained levels of CXCL10 in the serum, suggesting the potential for more durable Th1-type immune memory [23]. Flow cytometric analysis revealed that the number of monocytes recruited to the dLNs by liposomal QS-21 and CpG ODNs adjuvant was significantly higher than in the PBS group and the CpG ONDs-alone group (*p* < 0.05). Notably, the recruitment of neutrophils by the liposomal QS-21 and CpG ODNs adjuvant, was significantly higher than in all other groups (including groups with individual components), indicating that the synergistic effect of liposomal QS-21 and CpG ODNs adjuvant partly stems from its unique regulation of innate immune cell recruitment. Although neutrophils have traditionally been regarded as involved in early inflammatory clearance, recent studies have found that they can capture antigens and assist dendritic cells in antigen presentation by releasing neutrophil extracellular traps (NETs) [24,25]. Both the liposomal QS-21 and CpG ODNs adjuvant, and AS01_B_ significantly increased the expression levels of CD40 and CD86 co-stimulatory molecules on DCs, a key mechanism for effective T-cell activation. In vitro experiments confirmed that liposomal QS-21 and CpG ODNs adjuvant directly stimulated splenocytes to produce high levels of various cytokines and chemokines, including TNF-α, CCL3, CCL4, IFN-α, and IL-10, ruling out the possibility that these factors originated solely from tissue damage and directly demonstrating its ability to activate immune cells.

Previous research reported that CD169^+^ macrophages in lymph nodes are the primary target cells of liposomal QS-21 [26]. Stimulation of CD169^+^ macrophages by QS-21 activates the inflammasome, leading to their rapid depletion and the release of inflammatory factors such as caspase-1 and IL-1β. This subsequently drives the rapid recruitment of innate immune cells to the lymph nodes. This view was corroborated by inflammasome research [27], which also confirmed that the early inflammatory response in macrophages precedes the recruitment of innate immune cells. The experiments found that liposomal QS-21, CpG ODNs, and the liposomal QS-21 and CpG ODNs adjuvant, all caused an increase in the absolute counts of total macrophages in mouse dLNs, with a statistically significant difference only in the CpG ODNs group. However, liposomal QS-21 and the liposomal QS-21 and CpG ODNs adjuvant, caused a decrease in the absolute counts of CD169+ macrophages in mouse dLNs. Conversely, CpG ODNs significantly increased the absolute counts of CD169^+^ macrophages. Therefore, it is possible that, after stimulation by liposomal QS-21, CpG ODNs, and the liposomal QS-21 and CpG ODNs adjuvant, macrophages (including both CD169^+^ and CD169^−^ subsets) are recruited into the dLNs. Among these, CD169^+^ macrophages are the primary target of liposomal QS-21 and are thus rapidly depleted. Consequently, the net result for liposomal QS-21 or the liposomal QS-21 and CpG ODNs adjuvant stimulation is an increase in the absolute count of total macrophages in the lymph nodes, but a decrease in the absolute count of CD169^+^ macrophages, leading to a significant drop in the proportion of CD169^+^ macrophages among total macrophages. In contrast, CpG ODNs stimulation results in massive recruitment of macrophages into the lymph nodes, with both CD169^+^ and CD169^−^ subsets increasing substantially, so the proportion of CD169^+^ macrophages remain unchanged. The depleted CD169^+^ macrophages release inflammatory factors, promoting the rapid recruitment of DCs, neutrophils, and monocytes to the dLNs.

The composition of the liposomal QS-21 and CpG ODNs adjuvant, determines its unique mechanism of action. Its components, liposomal QS-21 and CpG ODNs, act both individually and synergistically. Liposomal QS-21 promotes antigen-specific humoral and cellular immunity, likely by influencing cell membrane permeability to enhance antigen uptake and presentation, while also potentially modulating immune cell function. CpG ODNs, as a specific TLR9 agonist, activates downstream signaling pathways to induce cellular and humoral immunity. Encapsulation of both components in liposomes likely facilitates in vivo transport and distribution, enhancing interaction with immune cells, thereby synergistically exerting a stronger immunostimulatory effect.

Certainly, this study has some limitations. Firstly, all experiments were conducted in healthy wild-type C57BL/6 mice, and the translation of these results to other animal strains or the clinic requires cautious evaluation. Secondly, although we preliminarily elucidated the cellular immune mechanisms of the liposomal QS-21 and CpG ODNs adjuvant, its detailed effects on germinal center B-cell reactions, long-lived plasma cells, and memory B/T cell generation remain to be clarified through more in-depth experiments (e.g., immunofluorescence microscopy analysis of germinal centers). Finally, important immune indicators such as mucosal immune responses were not evaluated, and the efficacy of liposomal QS-21 and CpG ODNs adjuvant with other important infectious disease or tumor antigens needs further assessment. Currently, multiple of our vaccine candidates utilizing this liposomal QS-21 and CpG ODNs composite adjuvant have advanced to Phase II clinical trials.

## 5. Conclusions

In summary, we have successfully developed a novel liposomal composite adjuvant. By synergistically utilizing the immunostimulatory pathways of saponin (QS-21) and a TLR agonist (CpG ODNs), this adjuvant achieves precise and potent modulation of the adaptive immune response, particularly favoring Th1-type immunity, which is crucial for clearing intracellular pathogens and combating tumors. Its potency is comparable to the clinically applied AS01_B_ adjuvant, and it demonstrates unique advantages in inducing key Th1-type chemokines. This study provides a promising adjuvant candidate and a solid experimental foundation for developing next-generation vaccines requiring potent cellular immunity, such as vaccines against shingles, tuberculosis, or therapeutic cancer vaccines. Future work will focus on evaluating its protective efficacy in challenge models and conducting systematic preclinical safety assessments.

## Figures and Tables

**Table 1:** Mice grouping and immunization schedule.

Groups	Number of Mice	Treatments (μg/per Mouse, 2 Doses: Week 0/3, i.m.)			
		gE	QS-21	CpG ODNs	MPL
PBS	6	/	/	/	/
gE	6	5	/	/	/
gE/QS-21	6	5	5	/	/
gE/MPL	6	5	/	/	5
gE/QS-21/MPL	6	5	5	/	5
gE/QS-21/CpG	6	5	5	10	/
gE/CpG/MPL	6	5	/	10	5
gE/QS-21/CpG/MPL	6	5	5	10	5

**Table 2:** Mice grouping and immunization schedule.

Groups	Number of Mice	Treatments (μg/per Mouse, 2 Doses: Week 0/2, i.m.)		
		gE	Liposomal QS-21	CpG ODNs
PBS	6	/	/	/
gE	6	2	/	/
gE/QS-21/CpG (1:1:10)	6	2	2	20
gE/QS-21/CpG (1:1:20)	6	2	2	40
gE/QS-21/CpG (1:0.5:5)	6	2	1	10
gE/QS-21/CpG (1:0.5:10)	6	2	1	20
gE/AS01_B_ (1/25 HD)	6	2	1/25 HD AS01_B_ (i.e., 2 μg liposomal QS-21 & 2 μg MPL)	

**Table 3:** Mice grouping and immunization schedule.

Groups	Number of Mice	Treatments (μg/per Mouse, 1 Dose, i.m.)		
		Liposomal QS-21	CpG ODNs	Liposomal QS-21/CpG ODNs
PBS	6	/	/	/
QS-21	6	2.5	/	/
CpG	6	/	25	/
QS-21/CpG	6	/	/	2.5/25

**Table 4:** Mice grouping and immunization schedule.

Groups	Number of Mice	Treatments (μg/per Mouse, 1 Dose, i.m.)		
		OVA	Liposomal QS-21/CpG ODNs	AS01_B_ (Liposomal QS-21/MPL)
OVA	3	20	/	/
OVA/QS-21/CpG	3	20	5/50	/
OVA/AS01_B_	3	20	/	5/5

**Table 5:** Mice grouping and immunization schedule.

Groups	Number of Mice	Treatments (μg/per Mouse, 1 Dose, i.m.)		
		OVA	Liposomal QS-21/CpG ODNs	AS01_B_ (Liposomal QS-21/MPL)
OVA	24	5	/	/
OVA/QS-21/CpG	24	5	5/50	/
OVA/AS01_B_	24	5	/	5/5

## Data Availability

All data used during the study are available from the corresponding author by request.

## Supplementary Materials

The following supporting information can be downloaded at: https://www.mdpi.com/article/10.3390/vaccines14060510/s1, Figure S1: Comparison of Hemolytic Activity between Free QS-21 and Liposomal QS-21; Figure S2: Gating plots of monocytes and neutrophils in flow cytometry experiments.
