# Supplementary material for: Development and Preliminary Mechanistic Evaluation of a Novel Liposomal QS-21 and CpG ODNs Adjuvant System for Enhancing Vaccine Immunogenicity
Source: Vaccines (Basel). 2026 Jun 5;14(6):510. doi: 10.3390/vaccines14060510 (PMC13307817; doi:10.3390/vaccines14060510)
Supplement: Supplementary file 1 [file vaccines-14-00510-s001.zip › vaccines-4316115-supplementary.pdf]

Supplementary Materials

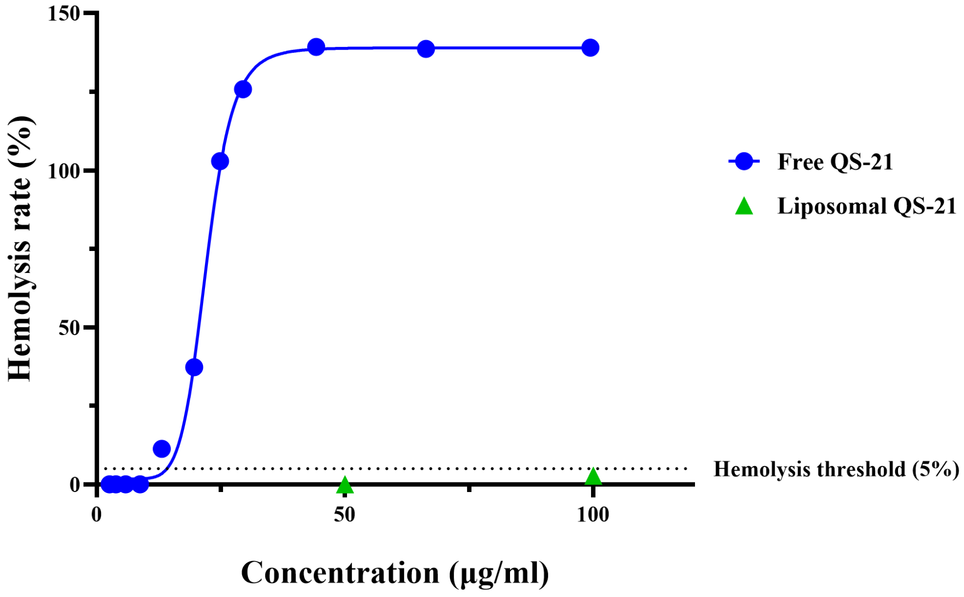

Figure S1. Comparison of Hemolytic Activity between Free QS-21 and Liposomal QS-21.

Free QS-21 exhibited a hemolysis rate exceeding 5% at a concentration of approximately 14 µg/ml. In contrast, liposomal QS-21 maintained a hemolysis rate of only 2.78% even at a much higher concentration of 100 µg/ml. These results demonstrate that liposomal encapsulation effectively mitigates the hemolytic toxicity of QS-21.

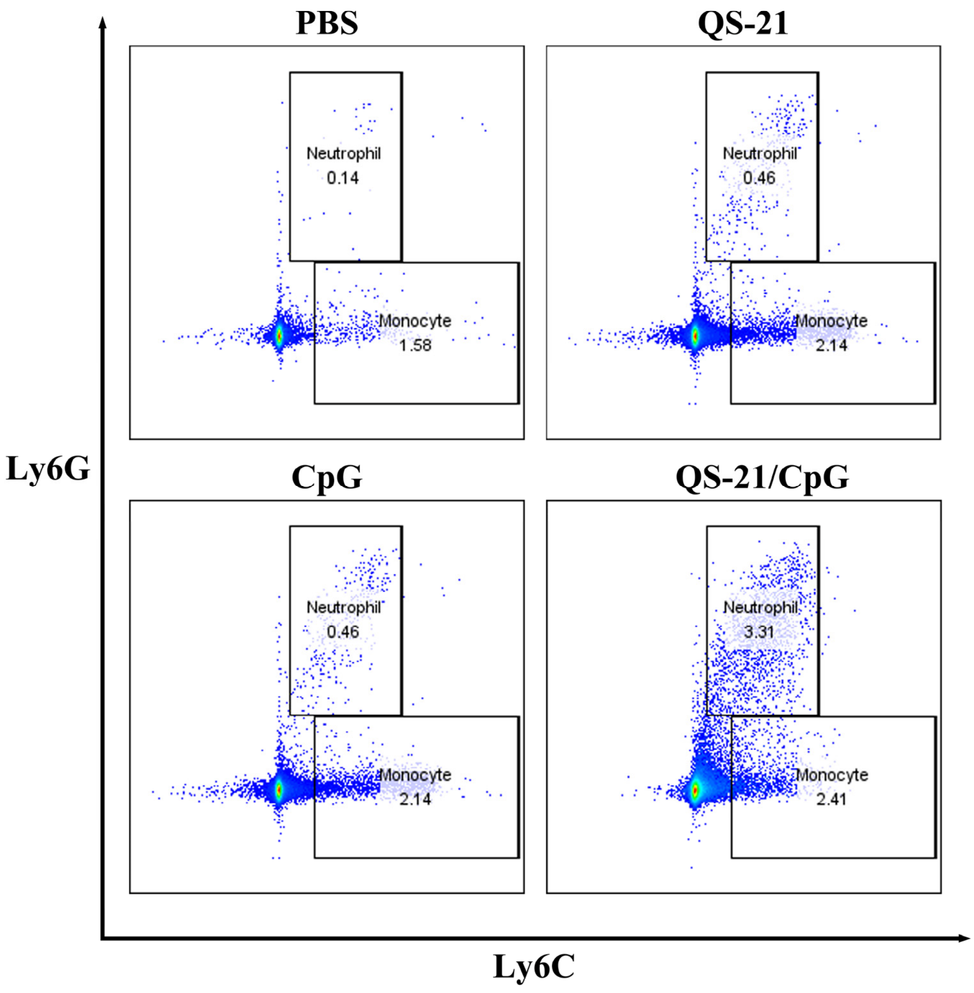

Figure S2. Gating plots of monocytes and neutrophils in flow cytometry experiments.
